# Osteopathic manipulative treatment and pain in preterms: study protocol for a randomised controlled trial

**DOI:** 10.1186/s13063-015-0615-3

**Published:** 2015-03-08

**Authors:** Francesco Cerritelli, Luca Cicchitti, Marta Martelli, Gina Barlafante, Cinzia Renzetti, Gianfranco Pizzolorusso, Mariacristina Lupacchini, Marianna D’Orazio, Benedetta Marinelli, Vincenzo Cozzolino, Paola Fusilli, Carmine D’Incecco

**Affiliations:** Clinical-based Human Research Department, C.O.ME. Collaboration, Via Amerigo Vespucci 188, 65126 Pescara, Italy; Accademia Italiana Osteopatia Tradizionale, Via Prati 29, 65124 Pescara, Italy; Neonatal Intensive Care Unit, Pescara’s public hospital, Via Fonte Romana 8, 65124 Pescara, Italy

**Keywords:** PIPP, Sham, Newborns, Cost, Placebo, Length of stay

## Abstract

**Background:**

Recent evidence proved the necessity to improve health care and pain management in newborns. Osteopathic manipulative treatment (OMT) has been largely used to treat painful syndromes as well as term and preterm newborns. Recent studies have demonstrated positive results of osteopathy in reducing length of stay and costs. However, no trials were carried out on pain in newborns. The aim of the present clinical trial is to explore the effectiveness of osteopathic treatment in reducing pain in a sample of preterms.

**Methods/design:**

A three-armed single blinded placebo-control randomised controlled trial protocol has been designed to primarily evaluate the extent to which OMT is effective in reducing pain in preterms. One hundred and twenty newborns will be enrolled from one tertiary neonatal intensive care unit in central Italy and randomised in three groups: study, sham and control. The study group will be further prospectively randomised in two subgroups: experienced osteopaths and students. All preterms will receive standard medical care. Osteopathic treatment will be applied to the study group only whilst ‘soft touch’ will be administer to the sham group only. Newborns will undergo manual sessions once a week for the entire period of hospitalisation. Blinding will be assured for neonatal staff and outcome assessor. Primary outcome will be the mean difference in baseline score changes of PIPP questionnaire between discharge and entry among the three groups. Secondary outcomes will be: mean difference in length of stay and costs between groups. Statistical analyses will use per-protocol analysis method. Missing data will be handled using last observation carried forward imputation technique.

**Discussion:**

The present single blinded randomised controlled trial has been designed to explore potential advantages of OMT in the management of newborns’ pain. Currently, based on a patient-centred need-based approach, this research will be looking at the benefit of osteopathic care rather than the efficacy of a specific technique or a pre-determined protocol.

**Trial registration:**

The protocol has been registered on ClinicalTrials.gov (NCT02146677) on 20 May 2014.

## Background

Worldwide public health policies are paying considerable attention to prematurity and neonatal outcomes [1]. To date, the prevalence of neonatal short-term and long-term complications are high [2] even more if prematurity is considered [3-5]. Interestingly, several studies have been published recently considering pain in neonates as one factor associated with immediate [6,7] and long-term consequences [3]. Laboratory research has confirmed these results, highlighting that early repeated tissue injuries alter somatosensory and nociceptive pathways producing central and peripheral sensitisation [8].

Recent evidence proved the necessity to improve health care and pain management in newborns [9]. During the last three decades, the attempt to assess and report pain in neonates has resulted in the development of dozens of measurements [9]. The majority of these tools based its assessment on behavioural, contextual and physiological indicators. However, for a large proportion of these instruments lack of construct validity and reliability are reported. The only tools which have also been largely validated in clinical settings are the Behavioural Indicators of Infant Pain Scale [10] and the Premature Infant Pain Profile (PIPP) [11]. The PIPP was consistently used in clinical neonatal settings and research context providing a reliable tool for neonatal pain assessment [12].

Osteopathic manipulative treatment (OMT) has been used to treat term and preterm newborns. Recent studies demonstrated the effectiveness of OMT in reducing length of stay (LOS) and costs [13] as well as the likelihood of gastrointestinal episodes [14]. Moreover, the absence of adverse events and side effects was widely reported, thus considering the approach to be safe [13,15].

OMT has been extensively used to treat, prevent and manage pain symptoms. Numerous research has been published addressing acute and chronic pain in different medical conditions [16-18]. However, no trials have been carried out on pain in newborns. The aim of the present clinical trial is to explore the effectiveness of OMT in reducing pain in a sample of preterms.

## Methods/design

The current protocol is designed for a single blinded placebo-control randomised controlled trial (RCT) aiming at evaluating the extent to which OMT is effective in reducing pain in preterms. Secondary objectives will be reduction of LOS and cost-effectiveness.

From 5 May 2014 to 30 September 2015 newborns will be enrolled in the study at one tertiary neonatal ICU (NICU). Newborns will enter and be randomised only after parents or guardians have given written informed consent.

Infants who are admitted to the NICU will be eligible for inclusion. Inclusion criteria are: being born in Pescara’s hospital, having a gestational age (GA) between 29 and 37 weeks, being of either gender and without clinical and/or congenital complications. Exclusion criteria will be applied as follows: lack of guardian consent, having a gestational age > 37 weeks, being a newborn with HIV and from HIV seropositive/drug-addicted mother, being transferred to/from other hospital, the presence of any congenital or genetical disease, neoplasms, neurological, cardiovascular, urinary, haematological abnormalities, proven or suspected necrotising enterocolitis or abdominal obstruction, birth trauma, surgical patients, pneumoperitoneum, atelectasis, respiratory distress.

### Randomisation and masking

**Figure 1:**
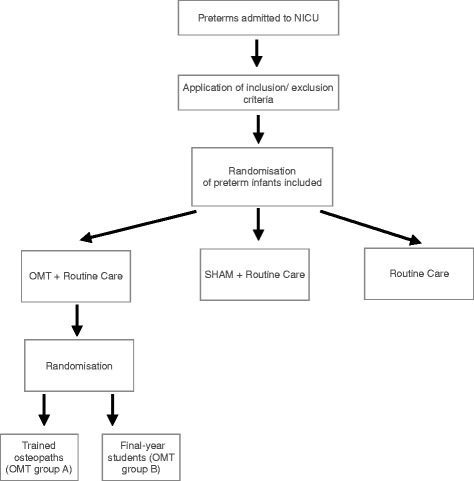
**Flow chart of the study.**

Patients will be randomly assigned in a 1:1:1 ratio to either the OMT, the sham or the control group (Figure 1). Newborns will be eligible for randomisation from the first day of life. Block randomisation will be performed according to a computer-generated randomisation list using a block size of nine, and stratified by OMT group.

Infants assigned to the study group will be further randomly divided into: OMT group A and OMT group B (Figure 1).

The coordinating centre will perform and store the randomisation list and an external consultant will be in charge of the process. NICU personnel will be unaware of outcome, study design and patients’ allocation. Only osteopaths will be aware of patients’ allocation.

### Osteopathic procedure

Osteopathic care includes an initial structural evaluation and a final treatment. The structural evaluation is carried out with the newborn lying down in bed and is aimed at diagnosing somatic dysfunctions [19]. The osteopathic manipulative treatment includes the application of a range of manipulative techniques aimed at treating the somatic dysfunctions. Techniques chosen for the present study are balanced ligamentous/membranous tension techniques [15]. The whole session will last 30 minutes: 10 minutes for evaluation and 20 minutes for treatment [15].

OMT will be performed by registered osteopaths with experience in the neonatology field (OMT group A) and final-year students (OMT group B). Newborns allocated to the study group will undergo osteopathic care in addition to usual care.

The sham group will receive only ‘soft touch’, for 30 minutes, plus routine care, whereas the control group will receive routine care only.

Osteopathic care and sham therapy will be administered once a week according to the treatment group. OMT groups A and B will enter the NICU on different days of the week.

### Data entering and data export

Data will be collected using an *ad-hoc* platform already used in previous studies [13,20,21]. Medical records will be acquired daily by the NICU staff.

Osteopathic records will be reported once a week when the osteopathic service is provided.

Maternal data will be also include collecting the following information: type of delivery, foetal presentation, whether placental abruption, pregnancy complications, single versus multiple gestation, whether premature rupture of membranes and any other complication.

Neonatal data will be gathered using the following parameters: gender, GA, birth weight, infants small for GA, neonatal complications (diagnosed at birth and during hospitalisation), diagnosis-related group (DRG) at discharge and reasons for drop out, if any.

A monitoring board will be organised and periodically will review the safety of data according to survival rate at discharge and relative risk of developing any clinical complication. The group sequential method will be utilised to define the rate at which type I errors occur; the Lan-DeMets generalisation of the O’Brien-Fleming boundary will be chosen. Interim analyses will be performed. Moreover, the monitoring board will be in charge of data export.

### Outcomes

Primary outcome will be the mean difference in baseline score changes of PIPP questionnaire between discharge and entry among the three groups.

Secondary outcomes will include:

- mean difference in PIPP questionnaire scores before and after each treatment
- mean difference in LOS measured in days between the three groups
- mean difference in costs between the three groups
- mean difference in PIPP questionnaire scores between the two OMT subgroups
- mean difference in LOS measured in days between the two OMT subgroups
- mean difference in costs between the two OMT subgroups
- difference in the risk of adverse events and side effects between the three groups
- average daily weight gain measured in grams

### Measures

The PIPP is a seven-item multidimensional (composite) measure of pain, largely used to assess acute pain in infants [11] and extensively validated for both preterm and term newborns [22]. Items are empirically and theoretically derived and are categorised as: contextual ((GA) and behavioural state (BS)), physiological (heart rate and oxygen saturation) and behavioural (facial actions). Numeric scores, reflecting baseline changes and based on an empirically 4-point scale (0, 1, 2, and 3), are provided for physiological and behavioural items. Conversely, the contextual items (GA and BS) have a reversed score (3, 2, 1, and 0) accounting for physiological differences related to prematurity. The maximum PIPP score is 21 for infants < 28 weeks GA and 18 for full-term newborns.

Considering LOS, according to international guidelines, well-determined discharge parameters are required and listed as follows: coordinated breathing, swallowing and sucking while feeding; stability of cardiorespiratory function; sustained weight gain and body heat at room temperature [23].

Regarding NICU costs, costs per day are calculated according to local authorities, multiplied by the days of hospitalisation. Costs are estimated in euros per day.

### Statistical analysis

According to Stevens *et al*. [11] the mean PIPP score for a painful procedure is 12.9 (standard deviation (SD), 3.4) and 6.0 (SD, 2.7) for a non-painful procedure. For a heel stick, the mean score is 10.3 (SD, 4.5) and 6.3 (SD, 3.2) for a simulated handling. However, as there is a lack of clinical trials in complementary medicine and/or manual disciplines, the sample size is computed considering an effect size (f) of 0.30, a statistical power of 0.80 and an α-level equal to 0.05 to produce a sample size of 37 per group. To prevent loss of power, the sample size is increased up to 40 subjects per group (total sample N = 120).

The primary analysis of the present clinical trial will be conducted according to per-protocol principle and intention-to-treat. Missing data will be handled using the last observation carried forward imputation technique. Arithmetic means and SD as well as median, percentage and range will be used to describe the general characteristics of the study population. Univariate statistical tests, Student’s *t*-test, analysis of variance (ANOVA) and chi square test, will be performed to compare the study groups and control group at the baseline. A linear mixed effect model will be used to evaluate the PIPP score across groups. Data will be analysed within and between the three study groups. Furthermore, a linear regression model will be applied to study the independent effect of OMT on primary and secondary endpoints between OMT groups A and B. Two-tailed *P*-values of less than 0.05 are considered to indicate statistical difference. To describe imbalances between groups, mean or relative risk with 95% CI will be used. Effect sizes will be computed stratifying by gestational age. R statistical programme (Vienna, Austria) will be used for data analysis [24].

### Cost analysis

Ordinary least squares regression will be performed to quantify the cost of hospitalisation among preterms after adjusting for gender, GA, LOS, birth weight, OMT, time to first OMT, and DRG. Cost data is extracted from 2014 to 2015 administrative databases of the Regional Office of the Ministry of Health of Abruzzo where the NICU of the present RCT is located.

To calculate the precise cost, the reimbursement allocated to each DRG from the Istituto Superiore di Sanità (National Healthcare Institute) is used. Specifically DRGs and reimbursements considered are 386 (€12.932.69), 387 (€7.450.09) and 388 (€3.757.22) [25].

As for the study group, OMT costs are theoretically set at €20.00 considering fees from health insurance companies [26].

Cost estimates will be adjusted for inflation to 2014 euro value using the Medical Component of the Consumer Price Index.

### Ethics

The approval of the Ethics Committee of the local health agency of Pescara (number 78/2013) has been obtained and the protocol has been registered on ClinicalTrials.gov (NCT02146677) on 20 May 2014. No disadvantages for the intervention are expected and no possible adverse events and side effects owing to OMT are predicted as confirmed by the recent osteopathic literature [13,15]. In any case, side effects and reason for drop out will be recorded during the research period and discussed in the final paper.

## Discussion

The present single blinded RCT has been designed to explore potential advantages of OMT in the management of newborns’ pain. Currently, based on a patient-centred need-based approach, this research will be looking at the benefit of OMT rather than the efficacy of a specific technique or a pre-determined protocol. Moreover, it will attempt to confirm the effectiveness of OMT on LOS-shortening using a larger experimental study design and to corroborate the absence of placebo effects in newborns. Furthermore, it will explore potential differences between expert osteopaths and final-year students on the effectiveness of OMT.

To date, no osteopathic trial has been carried out in this field attempting to quantify the benefits of OMT in newborns’ pain using rigorous procedures and ‘gold-standard’ methods for clinical trials. This will be the first concrete experimental trial addressing this outcome. The expected advantages from the present trial will be reduction of pain, shorter LOS and lower neonatal costs in the OMT groups compared to sham and control groups.

### Publication policy

The outcomes of the present research will be published in peer-reviewed journals and presented at congresses. The trial will be reported in accordance with the Consolidated Standards of Reporting Trials (CONSORT) recommendations.

## Trial status

The present protocol is for an ongoing research.

## Abbreviations

ANOVA: Analysis of variance
BS: Behavioural state
CONSORT: Consolidated standards of reporting trials
DRG: Diagnosis-related group
GA: Gestational age
LOS: Length of stay
NICU: Neonatal ICU
OMT: Osteopathic manipulative treatment
PIPP: Premature infant pain profile
RCT: Randomised controlled trial
